# Dementia incidence trend in England and Wales, 2002–19, and projection for dementia burden to 2040: analysis of data from the English Longitudinal Study of Ageing

**DOI:** 10.1016/S2468-2667(23)00214-1

**Published:** 2023-10-26

**Authors:** Yuntao Chen, Piotr Bandosz, George Stoye, Yuyang Liu, Yanjuan Wu, Sophia Lobanov-Rostovsky, Eric French, Mika Kivimaki, Gill Livingston, Jing Liao, Eric J Brunner

**Affiliations:** a Department of Epidemiology and Public Health, University College London, London, UK; b Division of Prevention Medicine & Education, Medical University of Gdansk, Gdansk, Poland; c Institute for Fiscal Studies, London, UK; d Department of Medical Statistics, School of Public Health, Sun Yat-sen University, Guangzhou, China; e Faculty of Economics, University of Cambridge, Cambridge, UK; f Division of Psychiatry, University College London, London, UK

## Abstract

**Background:**

Dementia incidence declined in many high-income countries in the 2000s, but evidence on the post-2010 trend is scarce. We aimed to analyse the temporal trend in England and Wales between 2002 and 2019, considering bias and non-linearity.

**Methods:**

Population-based panel data representing adults aged 50 years and older from the English Longitudinal Study of Ageing were linked to the mortality register across wave 1 (2002–03) to wave 9 (2018–19) (90 073 person observations). Standard criteria based on cognitive and functional impairment were used to ascertain incident dementia. Crude incidence rates were determined in seven overlapping initially dementia-free subcohorts each followed up for 4 years (ie, 2002–06, 2004–08, 2006–10, 2008–12, 2010–14, 2012–16, and 2014–18). We examined the temporal trend of dementia incidence according to age, sex, and educational attainment. We estimated the trend of dementia incidence adjusted by age and sex with Cox proportional hazards and multistate models. Restricted cubic splines allowed for potential non-linearity in the time trend. A Markov model was used to project future dementia burden considering the estimated incidence trend.

**Findings:**

Incidence rate standardised by age and sex declined from 2002 to 2010 (from 10·7 to 8·6 per 1000 person-years), then increased from 2010 to 2019 (from 8·6 to 11·3 per 1000 person-years). Adjusting for age and sex, and accounting for missing dementia cases due to death, estimated dementia incidence declined by 28·8% from 2002 to 2008 (incidence rate ratio 0·71, 95% CI 0·58–0·88), and increased by 25·2% from 2008 to 2016 (1·25, 1·03–1·54). The group with lower educational attainment had a smaller decline in dementia incidence from 2002 to 2008 and a greater increase after 2008. If the upward incidence trend continued, there would be 1·7 million (1·62–1·75) dementia cases in England and Wales by 2040, 70% more than previously forecast.

**Interpretation:**

Dementia incidence might no longer be declining in England and Wales. If the upward trend since 2008 continues, along with population ageing, the burden on health and social care will be large.

**Funding:**

UK Economic and Social Research Council.

## Introduction

More than 55 million people worldwide live with dementia, and this number is projected to increase threefold by 2050.^1^ Dementia is a multidimensional challenge with major implications for affected individuals, their families, social policy, and national economies. In England and Wales, the number of people living with dementia has been predicted to increase by 57% from 0·77 million in 2016 to 1·2 million in 2040.^2^ Corresponding health-care and social care cumulative costs have been projected to reach around £70 billion from 2020 to 2029.^3^ Although population ageing is causing an increase in worldwide dementia cases, this is partly offset by a declining dementia incidence trend in high-income countries.^4^, ^5^, ^6^, ^7^ If the incidence trend changed, the future burden of dementia could differ substantially from current forecasts, with major societal implications, particularly for care services.

Dementia incidence has apparently been declining in many high-income countries.^5^ Using population-based studies with consistent diagnostic measures, a declining age-specific incidence has been found in the USA,^7^, ^8^ UK,^2^, ^4^ Netherlands,^9^ France,^10^ and Sweden^11^, ^12^ (appendix pp 2–4). The sizes of estimated incidence declines are substantial, ranging from 12% to 35% per decade, although some studies do not find a downward trend (appendix pp 2–4).^13^, ^14^ However, these trend estimates mostly use data series ending in 2010 and there is little evidence on how the dementia incidence trend has evolved in the years since. Risk of dementia is inversely related to socioeconomic position in high-income countries.^15^, ^16^ Several studies have explored dementia incidence trends by education level, showing inconsistent results.^7^, ^12^, ^17^ We considered it informative to examine whether dementia incidence trend differed by education level.

**Research in context**

**Evidence before this study**

We searched the PubMed database for any studies published between Jan 1, 2010, and April 1, 2023, that estimate temporal trends in dementia incidence. The search terms used were: (trend[Title/Abstract] or trends[Title/Abstract]) AND ((“Dementia”[Mesh]) OR “Cognitive Dysfunction”[Mesh]) AND (“Incidence”[Mesh]), with no language restriction. We additionally hand-searched lists of references retrieved from relevant papers. A majority of studies in high-income populations found that dementia incidence decreased until 2010. Evidence on trends after 2010 was mainly based on studies using registry data or primary care data linkage with clinical diagnosis, which are likely to be affected by changes in diagnostic criteria and clinical practice over time. We found two US studies estimating the dementia incidence trend from 2000 to 2016 using data from the Health and Retirement Study, both of which made a linear trend assumption and thus implicitly assumed no change in the incidence trend during the period of observation. We did not identify any studies estimating the dementia incidence trend in the UK after 2010 using population-based data and a consistent diagnostic definition.

**Added value of this study**

The present study provides analyses of recent trends in dementia incidence in England and Wales to 2019. Using 18 years and nine waves of data from the English Longitudinal Study of Ageing, 2002–19, we showed that dementia incidence followed a non-linear trend. The rate declined by 28·8% from 2002 to 2008, followed by a 25·2% increase from 2008 to 2016. A similar non-linear pattern in dementia incidence was observed across subgroups according to age, sex, and educational attainment. There was a slower decline in 2002–08 and a faster increase after 2008 in participants with lower educational attainment. Using the point estimate for the recent upward trend of a 2·8% relative annual increase estimated in our study, the number of people with dementia in England and Wales is set to increase to 1·7 million by 2040, approximately twice the number in 2023.

**Implications of all the available evidence**

Most previous studies have shown that dementia incidence has declined in high-income countries. Our study indicates that dementia incidence started to increase in England and Wales after 2008. Inequalities in the dementia incidence trend were found to be widening between education groups. Continued monitoring of the incidence trend will be important in shaping social care policy. Based on the estimated upward incidence trend, we project that the number of people with dementia in England and Wales will be 1·7 million in 2040, indicating that the burden on health and social care might be considerably larger than current forecasts predict.

A methodological challenge in studies measuring dementia incidence is how to handle data for participants who develop dementia and then die between survey waves.^18^, ^19^ Most studies merely measure whether or not people have dementia in each observation period. Thus, they do not record people with newly incident dementia who die before the next follow-up, and therefore underestimate the dementia incidence. Even if dementia incidence does not change over time, differential bias over the period of observation due to changing mortality rates can produce a spurious upward or downward trend. For example, dementia incidence appeared to decline in the Framingham Heart Study over the period 1977–2008.^7^ However, a reanalysis correcting for this bias due to death, using the same three decades of follow-up data, did not replicate the decline.^20^

We used data from nine waves of the English Longitudinal Study of Ageing (ELSA) with a standardised algorithm-based dementia diagnosis to estimate the trend of dementia incidence in England and Wales from 2002 to 2019, considering bias and non-linearity. We aimed to explore whether the temporal trend of dementia incidence differed across age, sex, and educational attainment. A Markov model that embeds the trend in dementia incidence was used to project the future burden of dementia in England and Wales to 2040 under scenarios for a flatlining and an increasing incidence rate trend.

## Methods

### Study participants

ELSA is a longitudinal panel study of a representative sample of people aged 50 years or more living in private households in England.^21^ Data are collected using computer-assisted personal interviews and self-completion questionnaires, with additional nurse visits for the assessment of biomarkers every 4 years.^21^ We used all the available data spanning 17 years across wave 1 (2002–03) to wave 9 (2018–19). Refreshment samples were recruited at waves 3, 4, 6, 7, and 9 (appendix p 6). Mortality data were linked to participants who had provided written consent for linkage to official records from the National Health Service central register. Ethical approval for each one of the ELSA waves was granted by the National Research Ethics Service (London Multicentre Research Ethics Committee). All participants provided written informed consent.

### Case definition of dementia

In the primary analysis, dementia was defined by an algorithmic case definition based on coexistence of cognitive impairment and functional impairment, or a report of a doctor's diagnosis of dementia by the participant or caregiver. The algorithmic case definition follows DSM-IV and other clinical criteria in that it hinges on non-transient impairment in two or more cognitive domains, resulting in functional impairment^2^ (appendix p 5). With the application of stringent criteria requiring severe cognitive and functional impairment, this definition mainly captured moderate to severe dementia cases.

Considering that there was a sustained increase in medical awareness and rate of doctor diagnoses of dementia between 2005 and 2015,^22^ a sensitivity analysis was conducted using the algorithmic case definition only, excluding doctor diagnoses.

Cognitive impairment was defined as impairment in two or more domains of cognitive function tests applied to ELSA participants (such as orientation to time, immediate and delayed memory, verbal fluency, and numeracy function). Impairment in each domain of cognitive function was defined as a score of 1·5 SD or more below the mean compared with the population aged 50–80 years with the same level of education.^23^ To avoid the effect of transient cognitive decline, resulting from delirium or other mental disorders, if the participant improved by 1 SD or more on cognitive tests in the consecutive wave, they were considered to not have cognitive impairment. 9·8% of the participants with cognitive impairment were identified as having transient cognitive decline. For individuals unable to take the cognitive function tests, the Informant Questionnaire on Cognitive Decline was administered to a proxy informant (usually an immediate family member),^24^ and a score higher than 3·6 was used to identify cognitive impairment. The threshold used has both high specificity (0·84) and high sensitivity (0·82).^24^

Functional impairment was defined as an inability to carry out one or more activities of daily living independently, which included getting into or out of bed, walking across a room, bathing or showering, using the toilet, dressing, cutting food, and eating. For more detailed information about the definition of dementia, see Ahmadi-Abhari and colleagues^2^ and Guzman-Castillo and colleagues.^25^

### Statistical analysis

We created seven overlapping subcohorts with 4 years of follow-up (ie, 2002–06, 2004–08, 2006–10, 2008–12, 2010–14, 2012–16, and 2014–18), nested within the longitudinal ELSA cohort study to compute crude dementia incidence. In each subcohort, participants were included if they were 50 years of age or older and without dementia at baseline based on our definition. The follow-up time was from baseline to dementia diagnosis, death, or 4 years, whichever came first. We assumed date of dementia onset was the midpoint between the wave in which dementia was first reported or ascertained and the previous wave. We computed crude dementia incidence by dividing the number of incident cases by the number of person-years in each subcohort. To calculate standardised rates, we applied direct age and sex standardisation to England and Wales 2011 census population estimates. We computed crude dementia incidence in the same way for subpopulations divided by age (<75 *vs* ≥75 years), sex (men *vs* women), and educational attainment (no qualification *vs* O-level, A-level or equivalent *vs* university or higher). The incidence rate for the final wave 2018–19 could not be estimated because follow-up was not available to ascertain dementia cases. This limitation also applied to estimates obtained from the Cox model and multistate model below.

We estimated time trends in dementia incidence across 2002–19 in ELSA using two statistical approaches. First, we estimated the trend by fitting a Cox proportional hazards model with incident dementia as the outcome and terms for age, age squared, sex, interactions between age and sex, and calendar time. This approach, commonly conducted in previous studies,^5^, ^7^, ^26^ does not account for missing cases due to death bias. The Cox model combined data from all subcohorts to estimate the overall time trend. A restricted cubic spline function with internal knots at years 2006 and 2010 was used to identify a potential non-linear incidence trend across calendar years.^27^ A robust sandwich estimator was used to estimate the 95% CIs to account for non-independence.^28^ We checked the proportional hazards assumption by incorporating the interaction between time and calendar year and found the assumption was not violated.

Second, we fitted a three-state Markov model to estimate the time trend of dementia incidence (appendix p 7). We used all data in ELSA longitudinally in long format in the multistate model. We modelled three transitions—no-dementia to dementia, no-dementia to death, and dementia to death—as a function of age, sex, and calendar time. Age and calendar time were modelled as time-dependent variables. Sex was incorporated as a covariate. Therefore, each transition rate was specific to age, sex, and calendar time. Likelihood ratio tests determined whether relaxing the log-linearity assumptions of age via restricted cubic splines improved the model fit. We found that spline function improved the model fit and therefore modelled age with this more flexible specification. We checked the proportionality assumption by incorporating the interaction between age and calendar time, and found that the assumption was not violated. The calendar time coefficient for the transition from no-dementia to dementia was the outcome of interest, which we initially assumed to be constant (linear assumption) and subsequently explored for potential non-linearity via restricted cubic splines. Interaction between age and sex was incorporated in the model. Although transient cognitive decline was removed, we could not avoid misclassification of dementia status because of variability of cognitive function scores, especially when scores were close to cutoffs. A misclassification model was incorporated in the multistate model to account for potential misclassification. Our approach closely followed that of the msm package in R.^29^ Compared with the Cox model, the multistate model additionally accounted for missing dementia cases due to death bias.

The time trend of dementia incidence was estimated in subpopulations stratified by age, sex, and educational attainment using the multistate model, additionally incorporating an interaction term between calendar time and the subgroup variable. Likelihood ratio tests were conducted to evaluate whether the interactions were statistically significant.

We updated a validated Markov model, IMPACT-Better Ageing Model (BAM),^2^, ^25^ using nine waves of ELSA data from 2002 to 2019, which follows the progression of the England and Wales population aged 35 years or older across ten health states—characterised by presence or absence of cardiovascular disease (CVD), cognitive impairment, and functional impairment—from 2011 to 2040. Input data to inform IMPACT-BAM include the population structure, age–sex specific initial prevalence in 2011 of each health state in the model, and age–sex–calendar time specific transition probabilities between states. The model structure and methodology are presented in the appendix (pp 13–14). We used the updated IMPACT-BAM model, which embeds the latest trends of dementia incidence (estimated from the multistate model), to project the burden of dementia in England and Wales to 2040.

We considered the observed stalling progress in life expectancy in the UK after 2010. In the primary analysis, we projected cardiovascular and non-cardiovascular mortality up to 2040 using a two-dimensional P-spline approach^30^ with UK Office for National Statistics (ONS) mortality and population estimates for 2001–18 for England and Wales as inputs. Two alternative scenarios for mortality trends were conducted in the sensitivity analysis. First, we assumed a conventional counterfactual that mortality rates from 2018 would persist unchanged to 2040 (conservative assumption). Second, using ONS mortality and population estimates for 2001–18, we projected cardiovascular and non-cardiovascular mortality up to 2040 using Poisson regression assuming a log-linear association between calendar year and mortality (optimistic assumption).

We used R (version 4.1.2) for the statistical analyses. A p value less than 0·05 was considered to be statistically significant.

### Role of the funding source

The funder of the study had no role in study design, data collection, data analysis, data interpretation, or writing of the report.

## Results

Basic characteristics of eligible participants by survey year are presented in table 1. A total of 19 806 participants were included in the analysis. Participants in later waves were slightly older. The sex distribution was balanced across waves. In more recent waves, participants were more educated, and there were more participants with diabetes and fewer with CVD.

**Table 1:** Basic characteristics by survey year

		**Wave 2002 (N=11 523)**	**Wave 2004 (N=9154)**	**Wave 2006 (N=9339)**	**Wave 2008 (N=10 744)**	**Wave 2010 (N=10 090)**	**Wave 2012 (N=10 373)**	**Wave 2014 (N=9486)**	**Wave 2016 (N=8353)**	**Wave 2018 (N=8558)**
Age, years		6·51 (10·5)	66·1 (10·1)	65·3 (10·7)	65·6 (9·9)	67·0 (9·6)	67·0 (9·9)	67·7 (9·9)	69·1 (9·4)	68·2 (10·3)
Women		6293 (54·6%)	5073 (55·4%)	5131 (54·9%)	5884 (54·8%)	5567 (55·2%)	5693 (54·9%)	5237 (55·2%)	4626 (55·4%)	4748 (55·5%)
Men		5230 (45·4%)	4081 (44·6%)	4208 (45·1%)	4860 (45·2%)	4523 (44·8%)	4680 (45·1%)	4249 (44·8%)	3727 (44·6%)	3810 (44·5%)
Education										
	Low	5881 (51·0%)	4326 (47·3%)	3553 (38·0%)	4100 (38·2%)	3880 (38·5%)	3813 (36·8%)	3380 (35·6%)	2965 (35·5%)	2150 (25·1%)
	Middle	3064 (26·6%)	2584 (28·2%)	2824 (30·2%)	3255 (30·3%)	3025 (30·0%)	3225 (31·1%)	2974 (31·4%)	2581 (30·9%)	3066 (35·8%)
	High	2547 (22·1%)	2203 (24·1%)	2915 (31·2%)	3289 (30·6%)	3027 (30·0%)	3185 (30·7%)	2839 (29·9%)	2507 (30·0%)	3171 (37·1%)
BMI, kg/m^2^		NA	27·9 (4·9)	NA	28·3 (5·3)	NA	28·3 (5·3)	28·0 (5·3)	27·8 (5·2)	28·1 (5·2)
Hypertension		4355 (37·8%)	3944 (43·1%)	3928 (42·1%)	4230 (39·4%)	4159 (41·2%)	4135 (39·9%)	3772 (39·8%)	3361 (40·2%)	3364 (39·3%)
Diabetes		853 (7·4%)	788 (8·6%)	879 (9·4%)	1044 (9·7%)	1134 (11·2%)	1205 (11·6%)	1173 (12·4%)	1095 (13·1%)	1159 (13·5%)
Stroke		514 (4·5%)	469 (5·1%)	459 (4·9%)	488 (4·5%)	485 (4·8%)	498 (4·8%)	461 (4·9%)	473 (5·7%)	439 (5·1%)
CVD		1903 (16·5%)	1598 (17·5%)	1472 (15·8%)	1510 (14·1%)	1457 (14·4%)	1328 (12·8%)	1102 (11·6%)	1020 (12·2%)	984 (11·5%)

Data are n (%) or mean (SD). English Longitudinal Study of Ageing participants aged 50 years or more (race is 97% White). CVD=cardiovascular disease.

The age-standardised and sex-standardised rate of dementia declined from 2002 to 2010 (from 10·7 to 8·6 per 1000 person-years). The rate increased from 2010 to 2019 (from 8·6 to 11·3 per 1000 person-years; table 2). The non-linear trend (first decline, then increase) was consistent in subgroups divided by age, sex, and educational attainment. Using the algorithmic diagnosis only, dementia incidence followed a similar pattern (appendix p 9).

**Table 2:** Crude and standardised dementia incidence rates

		**2002–06**	**2004–08**	**2006–10**	**2008–12**	**2010–14**	**2012–16**	**2014–18**
Crude		8·7	9·1	8·4	7·4	7·4	8·7	10·3
Standardised*		10·7	11·1	10·6	10·3	8·6	10·2	11·3
Age								
	<75 years	4·2	3·9	3·1	3·5	3·7	4·1	4·9
	≥75 years	29·4	31·5	33·1	28·6	23·6	27·4	29·6
Sex								
	Women	8·1	8·9	9·1	7·7	6·7	8·3	10·2
	Men	9·4	9·4	7·5	7·2	8·2	9·2	10·3
Education								
	Low	10·0	11·6	11·9	9·9	9·5	10·8	12·8
	Middle	7·6	7·7	6·6	6·5	6·8	8·3	10·0
	High	7·0	6·0	6·1	5·4	5·5	6·6	7·6

English Longitudinal Study of Ageing, English Longitudinal Study of Ageing participants aged 50 years and over, 2002 to 2019. Crude dementia incidence rate estimated in seven 4-year subcohorts. Rates are per 1000 person-years.

* Age-standardised and sex-standardised rate using England and Wales 2011 Census population estimates.

**Figure 1:**
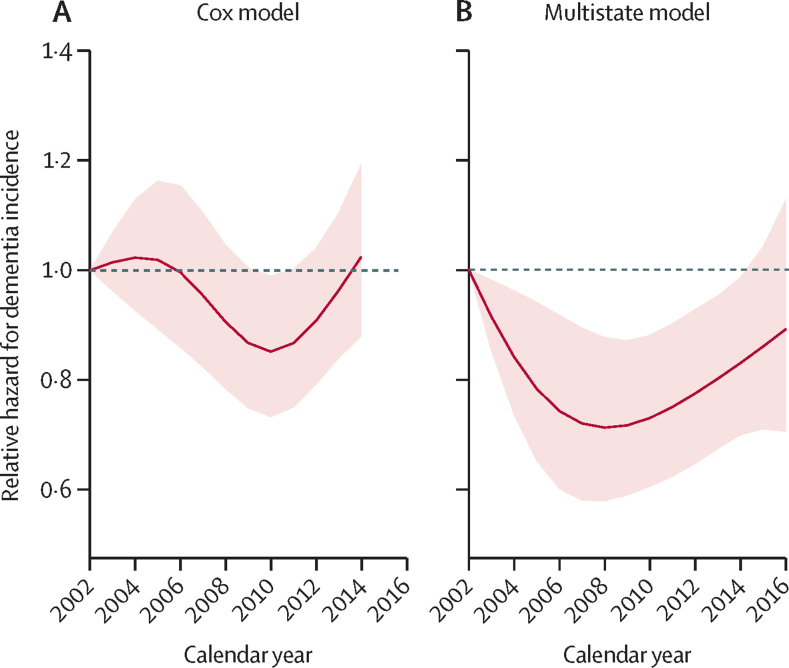
Age-adjusted and sex-adjusted dementia incidence rate ratio estimated from the (A) Cox model and (B) multistate model, 2002–19 Reference year 2002. Dashed lines represent 95% CIs. The multistate model (appendix p 7) accounted for potential bias due to deaths between waves among newly incident dementia cases. The incidence rate for the final wave 2018–19 could not be estimated. Cox model: hazard ratio 0·85, 95% CI 0·74–0·99 for year 2010 versus 2002, and 1·20, 1·04–1·39 for year 2014 versus 2010. Assuming a linear trend after 2010, dementia incidence increased by 4·6% annually. Multistate model: 0·71, 0·58–0·88 for year 2008 versus 2002, and 1·25, 1·03–1·54 for year 2016 versus 2008. Assuming a linear trend after 2008, dementia incidence increased by 2·8% annually.

The dementia incidence rate was estimated to follow a similar non-linear trend in both the Cox and multistate models (figure 1). In the Cox model, the age-adjusted and sex-adjusted dementia incidence rate declined by 14·7% from 2002 to 2010 (hazard ratio [HR] 0·85, 95% CI 0·74–0·99) and then increased by 19·9% from 2010 to 2014 (1·20, 1·04–1·39). In the multistate model, age-adjusted and sex-adjusted dementia incidence declined by 28·8% from 2002 to 2008 (incidence rate ratio 0·71, 0·58–0·88), followed by a 25·2% increase from 2008 to 2016 (1·25, 1·03–1·54). Using the algorithmic diagnosis only, dementia incidence followed a similar pattern with a slower upward trend after 2010 (appendix p 11).

**Figure 2:**
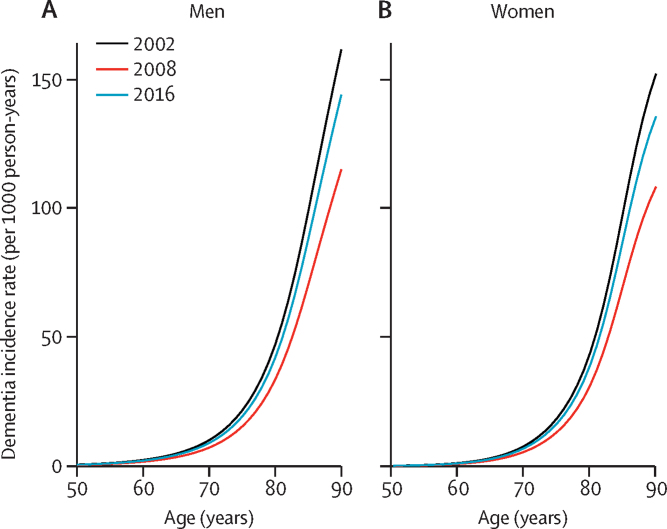
Dementia incidence rate by age in (A) men and (B) women at years 2002, 2008, and 2016 Rates were estimated from the multistate model using English Longitudinal Study of Ageing data, 2002–19.

Figure 2 shows the dementia incidence rate by age and sex in years 2002, 2008, and 2016 estimated from the multistate model. For men aged 80 years, the dementia incidence rate declined from 47·2 to 33·6 per 1000 person-years from 2002 to 2008, then rebounded to 42·1 per 1000 person-years in 2016. Similar patterns were observed for women and for different age groups.

**Figure 3:**
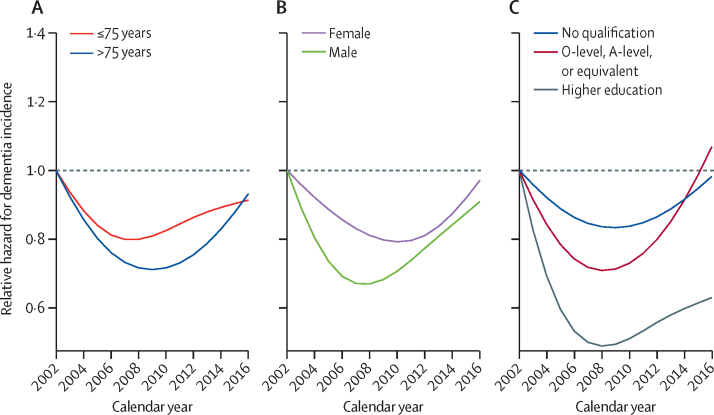
Age-adjusted and sex-adjusted dementia incidence ratio by (A) age, (B) sex, and (C) educational attainment Rates were estimated from the multistate model using English Longitudinal Study of Ageing data, 2002–19. Reference year 2002. p=0·78 for age, 0·84 for sex, and 0·0086 for educational attainment.

The dementia incidence trend showed a consistent non-linear pattern (first decline, then increase) in each subgroup. It did not differ by age group (p=0·78) or sex (p=0·84). The trend of dementia incidence differed significantly by educational attainment (p=0·0086). The group with higher educational attainment had a faster decline in dementia incidence from 2002 to 2008, and a slower increase after 2008 (figure 3). Using the algorithmic diagnosis only, dementia incidence followed a similar pattern (appendix p 12). The dementia incidence trend did not differ significantly by age, sex, or educational attainment, although a faster decline in the group with higher educational attainment was observed.

**Figure 4:**
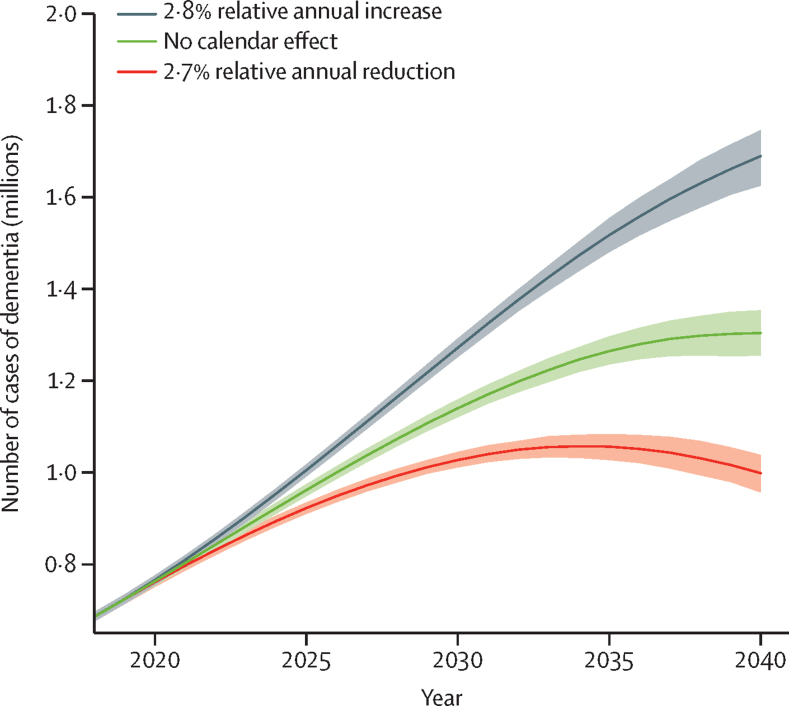
Projected number of people with dementia in England and Wales, 2018–40 Shaded areas represent 95% uncertainty intervals. 2·8% relative annual increase (black line) was estimated using English Longitudinal Study of Ageing data, 2002–19, based on a multistate model accounting for non-linear trend and bias. 2·7% relative annual reduction (red line) was based on a previous estimate using English Longitudinal Study of Ageing data, 2002–12.

Assuming dementia incidence continues to decline by 2·7% annually (as previously estimated^2^) after 2018, the number of people with dementia in England and Wales will increase from 0·7 million in 2018 to 1·0 million (95% CI 0·96–1·04) in 2040. However, assuming the upward trend of dementia incidence estimated from the multistate model in the present study (a 2·8% relative annual increase) would continue after 2018, the dementia projection is 1·7 million (1·62–1·75) in 2040, with 70% more dementia cases compared with the scenario with a downward trend (a 2·7% relative annual decrease). If dementia incidence remained constant after 2018, the forecast is that there would be 1·3 million (1·25–1·35) cases in 2040 (figure 4).

The dementia burden will rise over time regardless of whether we assume a conservative (mortality rates from 2018 would persist unchanged to 2040) or optimistic (mortality rates projected based on Poisson regression assuming a log-linear association between calendar year and mortality) mortality trend scenario (appendix p 18). The predicted number of people with dementia in 2040 is larger in the optimistic mortality scenario, because in this scenario people will have more years of life and will thus spend a larger share of their life in old age, when dementia incidence is especially high. Consistently, there are 70% more dementia cases with an upward compared to a downward incidence trend.

## Discussion

Our longitudinal study suggests that the decreasing time trend in dementia incidence reversed in 2008. Using the central estimate for the recent upward trend, the number of people with dementia in England and Wales is predicted to increase to 1·7 million by 2040, approximately twice the number in 2023.

We used population-representative cohort data with a consistent algorithm-based dementia diagnosis. The modelling method took account of potential bias in the trend estimate through underascertainment of dementia cases due to mortality risk between survey waves. We found that dementia incidence followed a non-linear trend. It declined by 28·8% from 2002 to 2008, followed by a 25·2% increase from 2008 to 2016. The decline and subsequent rise in dementia incidence was observed across all age, sex, and education subgroups. Consistent with views that dementia risk is modifiable, there was a sharper decline in 2002–08 and a slower increase after 2008 in participants in the group with higher educational attainment.

A majority of recent studies in high-income populations have shown that dementia incidence has decreased over the last two decades (appendix pp 2–4). Our finding is consistent with these studies, and confirmed our previous finding^2^ of a decline in the trend of dementia incidence in England and Wales in the ten years since 2002, using robust modelling methods. Our previous finding was based on the first six waves (2002–12) of the ELSA study, assuming a linear dementia incidence trend. The present study included nine waves (2002–19) of ELSA, enabling us to estimate the trend of dementia incidence in the most recent decade. Importantly, allowing for a non-linear trend of dementia incidence, we observed a pattern of increasing dementia incidence after 2010, which might be a signal of a qualitative change in the long-term trend. Little evidence has been reported for the trend of dementia incidence since 2010. A study using Global Burden of Disease 2019 data showed that the rate of dementia incidence in the UK did not decline from 2010 to 2019 (8·30 *vs* 8·29 per 1000 person-years).^31^ However, the Global Burden of Disease data might not provide a reliable estimate of the dementia incidence trend, because the estimate uses multiple data sources that cover different time periods and use different case ascertainment methods.

The reversal of the trend of dementia incidence occurred together with the stalling in UK mortality rates. The underlying explanation for their coincidence might be chance. However, obesity and type 2 diabetes are shared risk factors for both dementia and mortality. The prevalence of adult obesity rose rapidly from 1975 to 2000,^32^ and this epidemic might have contributed to increasing dementia incidence in recent cohorts of older people.^33^, ^34^ Other possible explanations include worsening risk factors in socially disadvantaged groups^35^, ^36^, ^37^, ^38^, ^39^ and improved survival for patients with stroke.^40^ If our finding on the reversal of the dementia incidence trend is replicated in other high-income countries, a systematic research effort is needed to explain this epidemiological pattern. Current evidence is that the recognised set of 12 modifiable risk factors explains approximately 40% of dementia cases worldwide at most.^39^

Subgroup analysis revealed widening inequalities in dementia incidence across education groups. People with lower educational attainment had a slower decline in dementia incidence from 2002 to 2008 and a faster increase after 2008. This finding might be explained by socioeconomic inequalities in dementia risk factors, such as midlife obesity.^38^ Similarly, a study in Shanghai observed a stable trend of dementia incidence in participants with more than 6 years of education, but an increasing trend in those with 6 years or less of education.^41^ In contrast, a Stockholm-based longitudinal study observed a higher reduction in dementia incidence in people with low educational attainment.^12^

The underlying dementia incidence trend is a key determinant of the future disease burden. If the upward trend evident for the period 2008–19 continues in the future, the burden will increase in England and Wales to 1·7 million in 2040, with 70% more people living with dementia compared with the scenario with the previously estimated downward trend.^2^ Changes in mortality rates are another important determinant of the number of people with dementia in the future, as they are associated with both life expectancy and the pool of individuals susceptible to dementia. Our previous study projected future cardiovascular and non-cardiovascular mortality using the Bayesian Age Period Cohort model with ONS mortality and population estimates for 1982–2012 as inputs.^2^ The mortality projection was overoptimistic as mortality improvements have stalled since 2010.^37^ In this study, we used more recent ONS mortality and population estimates for 2001–18 to project future mortality rates. We compared how different mortality trend scenarios would impact the future dementia burden and found that there would be consistently around 70% more cases with the upward incidence trend we estimated compared with the previously estimated downward incidence trend in all the mortality trend scenarios. The dementia incidence trend appears to be a more important determinant of future dementia burden compared with the mortality trend, and public health policies to reverse the upward incidence trend are needed urgently to mitigate the large burden of dementia in the future.

We used a representative sample of the population aged 50 years or more living in private households using a consistent standardised dementia case definition. The assessment criteria are consistent across time and thus more informative of dementia trends than clinical assessments, which are likely to be affected by changes in diagnostic criteria and clinical practice over time.^10^, ^22^, ^42^

An important methodological strength relative to previous studies is accounting for missing dementia cases due to death. This bias is different from the issue of competing risks of death. Commonly, absolute risk or cumulative incidence of dementia can be calculated based on a competing risk framework such as the Fine-Gray model, in which the rate of dementia is considered in people who are either currently dementia-free or who previously died.^43^ These methods are biased and should not be applied in this study, because we are interested in the instantaneous rate of dementia in living people who are currently dementia-free. Our approach using a multistate model (appendix p 7) accounts for a different bias due to participants who develop dementia and then die before the subsequent wave of data collection.^18^, ^19^ Another way to address this bias is to incorporate information from medical records and death causes through linkage to hospital episode statistics and death registers. However, this is likely to introduce bias because of changes in diagnostic criteria and clinical practice over time. To address this issue, we used a multistate model and found that the drop in dementia incidence before 2008 was larger and the rise after 2008 was smaller (figure 1) than in the findings using a Cox model, which did not account for this problem. Addressing bias from missing information because of death is important because a small change in the dementia incidence trend will result in a substantially enlarged difference in future dementia burden.

The direction of the bias in this study is different from that in the Framingham reanalysis, in which the use of multistate model led to the inference of no decline in dementia incidence. In the Framingham reanalysis, violation of the proportional hazards assumption in the Cox model could be an important reason for its inconsistent results.^20^ This assumption was not violated in our study. Additionally, differing mortality trends in different periods and populations can produce bias in an opposite direction.^18^, ^19^

ELSA participants were not clinically screened for dementia. Rather, our case definition for dementia is based on repeated standardised cognitive and functioning assessments, and follows DSM-IV and other clinical criteria in that it hinges on non-transient impairment in two or more cognitive domains, resulting in functional impairment. Cognitive assessment in ELSA is based on a set of validated cognitive function tests. Cognitive impairment in domains other than those tested may have been missed, leading to underestimation of dementia cases. A previous study found that age-specific and sex-specific incidence rates of dementia using this case definition were in line with those obtained in other European studies, including the populations of England (CFAS-II), Italy (Italian Longitudinal Study of Ageing), and Spain (NEDICES study), but higher than those in the Rotterdam study and lower than those in American populations of Minnesota (Mayo Clinic Study of Ageing) and White participants in the Cardiovascular Health Study (1989–99).^2^ Although not suitable in a clinical setting, the similarity of our estimates for age-specific dementia incidence to those of independent studies underpins the validity of our case definition for dementia at population level. To date, we are not aware of any population-based studies in the UK that have age-specific dementia incidence and prevalence estimates using a clinical adjudication process. Harmonized Cognitive Assessment Protocol, a neuropsychologist interview-based substudy in ELSA, will provide this evidence in the future.^44^ Crucially, the consistent algorithmic case definition across the ELSA waves provides a reliable estimate for time trends even if it might lead to underestimation or overestimation of dementia incidence in a particular year. The proportion of ethnic minority participants in ELSA was very small, and our results might not be generalisable to those minorities. Survey and non-response might bias the estimate for dementia incidence trend. Sensitivity analysis in which survey weights were applied showed a similar time trend (appendix p 19). Although model assumptions including proportionality and linearity were carefully checked, we cannot preclude potential bias due to model misspecification.

We did not take into account potential future changes in other dementia risk factors in the IMPACT-BAM Markov predictions. Our modelling approach is to incorporate trends of mortality and disease incidence that reflect the composite trend of risk factors. The Markov model provides a platform to examine the impact of individual and multiple risk factor changes on the future burden of dementia through scenario modelling,^3^, ^45^, ^46^ beyond the scope of the present study. We include CVD death and non-CVD death states in the IMPACT-BAM model because the former is the main driver of the reduction of all-cause mortality (appendix p 17), in addition to its role as a determinant of dementia prevalence. Competing risks due to death from non-CVD causes, such as cancer and COPD, are accounted for through an aggregated non-CVD death state.

In conclusion, dementia incidence followed a non-linear trend in England and Wales with a declining trend from 2002 to 2008 and an increased trend from 2008 to 2016. Inequalities in dementia incidence trend widened between education groups. If the upward incidence trend continues, along with population ageing, the number of people with dementia in England and Wales is projected to increase to 1·7 million in 2040. Continued monitoring of the incidence trend will be important in shaping social care policy. The burden on health and social care might be considerably larger than currently forecast.

## Data sharing

The data involved in this analysis are available through the UK Data Service (through which data from ELSA [elsa-project.ac.uk] can be accessed). A technical appendix with details on the formulae and calculations for the IMPACT-BAM model is available from the corresponding author.

## Declaration of interests

The authors declare no competing interests.
