# Supplementary material for: Dementia incidence trend in England and Wales, 2002–19, and projection for dementia burden to 2040: analysis of data from the English Longitudinal Study of Ageing
Source: Lancet Public Health. 2023 Oct 26;8(11):e859–67. doi: 10.1016/S2468-2667(23)00214-1 (PMC10958989; doi:10.1016/S2468-2667(23)00214-1)
Supplement: Supplementary appendix [file mmc1.pdf]

THE LANCET
Public Health

Supplementary appendix

This appendix formed part of the original submission and has been peer reviewed. We post it as supplied by the authors.

This online publication has been corrected. The corrected version first appeared at thelancet.com/public-health on Jan 2, 2024

Supplement to: Chen Y, Bandosz P, Stoye G, et al. Dementia incidence trend in England and Wales, 2002–19, and projection for dementia burden to 2040: analysis of data from the English Longitudinal Study of Ageing. *Lancet Public Health* 2023; **8:** e859–67.

Supplementary Materials

Dementia incidence trend in England and Wales, 2002-2019, and projection for dementia burden to 2040: analysis of data from the English Longitudinal Study of Ageing

Yuntao Chen[a] PhD, Piotr Bandosz[b] PhD, George Stoye[c] PhD, Yuyang Liu[d] MSc, Yanjuan Wu[d] MSc, Sophia Lobanov-Rostovsky[a] MSc, Prof Eric French[e] PhD, Prof Mika Kivimaki[f] PhD, Prof Gill Livingston[f] MD, Jing Liao[d] PhD, Prof Eric J Brunner[a] PhD

[a] Department of Epidemiology and Public Health, University College London, London, UK
[b] Division of Prevention Medicine & Education, Medical University of Gdansk, Gdansk, Poland
[c] Institute for Fiscal Studies, London, UK
[d] Department of Medical Statistics, School of Public Health, Sun Yat-sen University, Guangzhou, China
[e] Faculty of Economics, University of Cambridge, Cambridge, United Kingdom
[f] Division of Psychiatry, University College London, London, UK

Corresponding author:
Yuntao Chen
Department of Epidemiology and Public Health, University College London, UK
1-19 Torrington Place
London WC1E 7HB
Email address: yuntao.chen@ucl.ac.uk

Table S1 Literature review of the trend of dementia incidence

	Data and Country	Period assessed	Methods	Estimates	Dementia diagnosis criteria
Beerten et al. BMJ open 2022[1]	Primary care practices across Flanders, Belgium	2000-2021	Jointpoint regression model	Increase from 2000 to 2018 (APC: 1·8% 95%CI -2·0 to 5·7%) and decrease from 2018 to 2021 (APC -8·1%, -14·8 to -0·8%)	Clinical diagnosis by linkage to electronic health records
Hegelund et al. Alzheimers Dement. 2022[2]	Register study in Denmark	2005-2018	Age- and sex-standardized incidence rates	Decrease by 17·3% per decade for men and 26.3% per decade for women	Clinical diagnosis (ICD-10)
Farina et al. J Aging Health. 2022[3]	Health and Retirement Study in US	2000-2016	Markov-based logistic model	Decrease by 2% annually (OR 0·98, 0·97 to 0·99)	Cognitive test performance or proxy scores
Bohlken et al. J Alzheimers Dis. 2021[4]	General and specialists' practices in Germany	2015-2019	Crude incidence rate	Decrease from 2015 to 2019 (0·44% to 0·35%)	Clinical diagnosis (ICD-10)
Ding et al. Alzheimers Dement 2020[5]	Two population-based cohort study in Shanghai, China	1987-1992<br>2010-2016	Logistic model	Increasing trend (OR 1·96, 1·40 to 2·75)	DSM-III for old cohort and DSM-IV for recent cohort
Ding et al. Alzheimers Dement 2020[6]	Two population-based cohort study in Kungsholmen, Sweden	1987-1998<br>2001-2013	Cox model	Declining trend (HR 0·70, 0·61 to 0·80)	DSM-III-R for older cohort and DSM-IV for recent cohort
Power et al. JAMA Neurol 2021[7]	Health and Retirement Study in US	2000-2016	Cox model	Results are mixed<br>HR_1 0·97, 0·89 to 1·05;<br>HR_2 0·88, 0·79 to 0·97;<br>HR_3 0·93, 0·85 to 1·03	Three previously developed and validated algorithms
Liu et al. Biomed Res Int 2019[8]	National Health Insurance Claim data in Taiwan	2004-2010	Poisson regression	Stable	Clinical diagnosis (ICD-9-CM)
Kirson et al. J Am Geriatr Soc 2020[9]	Medicare beneficiaries in US	2007-2014	Crude incidence rate	Decrease from 2007 to 2014 (1·53% to 1·09%)	Clinical diagnosis of AD (ICD-9)
Vanderkruk et al. Dement Geriatr Cogn Disord 2020[10]	Population-based retrospective cohort study in Ontario, Canada	2010-2015	Age- and sex-standardized incidence rate	Decrease by 15·8% over 6 years (3·9 to 3·3 per 1000 population)	Clinical diagnosis from various data sources
Wolters et al. Neurology 2020[11]	Population-based cohort study in Europe and US	1988-2015	Cox model	Decrease by 13% per decade (95%CI 7-19%)	DSM-III-R or DSM-IV
Taudorf et al. Alzheimers Dement 2019[12]	Registry data in Denmark	1996-2015	Poisson regression	Increase by 9% annually until 2003, followed by 2% annual decline	Clinical diagnosis (ICD-10)

Petersen et al. J Alzheimers Dis 2019[13]	Registry data in Faroe Islands, Denmark	2010-2017	Poisson regression	Fluctuate between 4·8 and 6·7 per 1000 population, stable	Clinical diagnosis (ICD-10)
Rajan et al. Alzheimers Dement 2019[14]	Prospective population-based study in Chicago, US	1994-2012	Weighted logistic model	Fluctuate	Uniform clinical evaluation (NINCDS-ADRDA criteria)
van den Kommer et al. J Gerontol B Psychol Sci Soc Sci 2018[15]	Population-based study in the Netherlands	1992-2016	Generalized estimating equations	Increase by 3.5% annually (OR 1·035, 1·015 to 1·055)	Consistent diagnosis for persistent cognitive decline using MMSE and IQCODE
Donegan et al. Lancet Public Health 2017[16]	CPRD practice data in the UK	2005-2015	Binomial generalised linear model	Doubled from 2005 to 2015 (0·42% to 0·82%)	Clinical diagnosis
Ahmadi-Abhari et al. BMJ 2017[17]	Population-based study in England (ELSA wave 1-6)	2002-2012	Joint modelling	Decrease by 2·7% annually (OR 0·973, 0·971 to 0·976)	Consistent diagnosis using cognitive and function impairment, and self-reported doctor diagnosis
Ohara et al. Neurology 2017[18]	Two population-based studies in Hisayama, Japan	1988-1998<br>2002-2012	Cox model and Fine-Gray model	Increase by 3·8% and 4·2% annually	Two-stage examination (first HDS-R, then other records including medical records)
Gao et al. Age Ageing 2017[19]	Population-based study in China (CLHLS)	1998-2014	Two-level poisson regression	Decrease (58·8 to 10·1 per 1000 population from 1998 to 2014)	CMMSE < 18 for cognitive impairment
Cerasuolo et al. Alzheimers Dement 2017[20]	Administrative data in Ontario, Canada	2002-2013	Crude incidence rates	Stable in men and decrease in women (-6·74% per decade)	Clinical diagnosis (ICD-9)
van Bussel et al. PLoS Med 2017[21]	GP registry data in the Netherlands	1992-2014	Negative binomial regression analysis	Increase by 2·1% annually (IRR 1·021, 1·005 to 1·038)	International Classification of Primary Care code P70
Noble et al. J Alzheimers Dis 2017[22]	Two cohort studies in Manhattan, US	1992-1994<br>1999-2001	Cox model	Decrease by 41% over 7 years (HR 0·59, 0·49 to 0·70)	DSM-IV
Satizabal et al. N Engl J Med 2016[23]	Framingham Heart Study in US	1977-2008	Cox model	Decrease by 20% per decade (HR 0·80, 0·72 to 0·90)	DSM-IV
Matthews et al. Nat Commun 2016[24]	Two population-based studies in the UK: CFAS I and II	1991-1993<br>2008-2011	Poisson regression	Decrease by 20% (IRR 0·8, 0·6 to 1·0, p=0·08)	Algorithmic diagnosis (GMS-AGECAT)
Grasset et al. Alzheimers Dement 2016[25]	Two population-based cohorts in Bordeaux, France	1988-1998<br>1999-2009	Multistate model	Stable for clinical diagnosis (HR 0·92, 0·73 to 1·15); decrease by 35% per decade (HR 0·65, 0·53 to 0·81)	Clinical diagnosis; Consistent algorithmic diagnosis based on cognitive and functional assessments

Kosteniuk et al. Int Psychogeriatr 2016[26]	Administrative data in Saskatchewan, Canada	2005-2013	Age-standardized incidence rate	Decrease by 1·4% annually	Clinical diagnosis (ICD-10)
Doblhammer et al. Alzheimers Res Ther 2015[27]	Health claims data in Germany	2004-2007 2007-2010	Proportional hazards model	Decrease by 3·1% annually	Clinical diagnosis (ICD-10)
Jorgensen et al. Eur J Public Health 2015[28]	Registry data in Denmark	2000-2009	Jointpoint regression model	Increase from 2000 to 2003, followed by stagnation until 2009	Clinical diagnosis for Alzheimer's disease
Qiu et al. Neurology 2013[29]	Two population-based cohort study in Kungsholmen, Sweden	1987-1994 2001-2008	Incidence trend can be inferred by prevalence and survival trend	Declining	DSM-III-R
Schrijvers et al. Neurology 2012[30]	Rotterdam study in the Netherlands	1990-1995 2000-2005	Poisson regression	Decrease by 25% over 10 years (IRR 0·75, 0·56 to 1·02)	DSM-III-R
Rocca et al. Alzheimers Dement 2011[31]	Community-based studies and HRS in US	1975-1994 1997-2008	Poisson regression; logistic model	Stable	Medical record linkage, clinical diagnosis

Case definition of dementia in relation to DSM-IV criteria
(Note this part reproduced from Ahmadi-Abhari et al., 2017 supplementary material[17]**)**

We adapted the dementia case definition to resemble DSM-IV and other criteria (such as NINDES-AIREN and NINCDS-ADRDA) for diagnosis of dementia. The cornerstone of clinical diagnostic criteria for dementia is impairments in two or more cognitive domains that result in considerable loss of function. Thus, we defined cognitive impairment as a score of equal to or lower than 1.5 standard deviations below mean, standardized to the population aged 50-80 with the same level of education, similar to criteria used for defining cognitive impairment no dementia (CIND).1 Loss of function was defined as impairments in conducting activities of daily living. We sought with a set of criteria to encompass all types of dementia and not merely Alzheimer's disease. Although memory impairment is a key element in the diagnosis of Alzheimer's disease, memory is affected to varying degrees in vascular, fronto-temporal and Lewy body types of dementia. Thus, memory impairment was not included as a necessary criterion in defining cognitive impairment in this study.

DSM-IV criteria specify that the disturbances do not occur exclusively during the course of delirium and are not better accounted for by another mental disorder. For the criteria to hold, and to increase specificity, transient impairments in cognitive function or conducting ADLs, were not classified as cognitive or functional impairment. Inclusion of impairments in conducting instrumental activities of daily living in case definition of dementia would have increased the sensitivity of our case-definition and would have enabled us to identify mild cases of dementia as well as the moderate to severe cases. However, this would also result in a great number of false positives. To ensure high specificity and to obtain unbiased estimates, we applied stringent criteria, requiring severe cognitive and functional impairment, for classification as dementia. As a result, only moderate to severe dementia cases are included in this study.

Table S2 English Longitudinal Study of Ageing waves and data collection

Year	Sample	Refreshment	Response rate
Wave 1 (2002/03)	12100		66%
Wave 2 (2004/05)	9432		82%
Wave 3 (2006/07)	9771	HSE 2001/02/03/04, Age 50-52	73%
Wave 4 (2008/09)	11050	HSE 2006, Age 50-74	74%
Wave 5 (2010/11)	10274		80%
Wave 6 (2012/13)	10601	HSE2009/10/11, Age 50-55	78%
Wave 7 (2014/15)	9666	HSE2011/12, Age 50-51	77%
Wave 8 (2016/17)	8445		82%
Wave 9 (2018/19)	8736	HSE 2013/14/15, Age 50-51	80%

HSE: Health Survey for England; Wave 1: original sample interviewed in HSE 1998/1999/2001, age 50+ on 1 March 2002. Response rate at a given wave are calculated by dividing the total number of respondents by the total number of individuals deemed eligible for that wave.

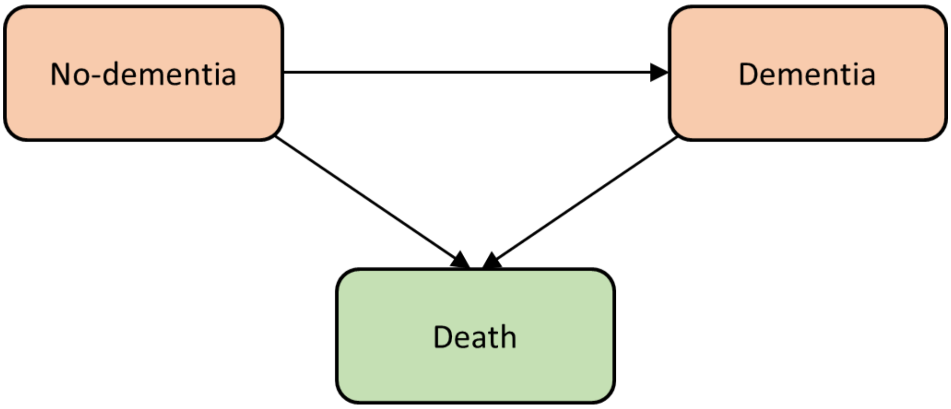

Figure S1. The multi-state Markov model

Table S3 Crude dementia incidence rate

	2002-2006	2004-2008	2006-2010	2008-2012	2010-2014	2012-2016	2014-2018
**Event**	371	305	284	291	269	324	341
**Pearson years**	42528	33432	33895	39071	36361	37305	33227
**Crude rate**	8·7	9·1	8·4	7·4	7·4	8·7	10·3

English Longitudinal Study of Ageing, aged 50 and over, 2002 to 2019. Crude dementia incidence rate estimated in seven 4-year subcohorts. Rates are per 1000 person-years.

Table S4 Crude and standardised dementia incidence rate

	2002-2006	2004-2008	2006-2010	2008-2012	2010-2014	2012-2016	2014-2018
**Crude**	7·8	8·0	7·1	6·2	6·1	6·4	7·3
**Standardised***	9·8	9·8	9·0	8·5	7·0	7·4	8·0
**Age, years**							
<75	3·8	3·5	2·5	2·7	3·0	2·8	3·4
≥75	26·5	27·0	28·2	24·3	19·6	20·5	21·2
**Sex**							
Women	7·3	7·5	7·6	6·3	5·4	5·8	6·9
Men	8·4	8·5	6·4	6·1	6·9	7·0	7·9
**Education**							
Low	8·7	9·7	9·1	7·6	7·8	7·8	8·7
Middle	7·4	7·1	5·9	6·0	6·4	6·1	6·8
High	6·4	5·8	5·8	4·7	3·7	4·9	6·4

English Longitudinal Study of Ageing, aged 50 and over, 2002 to 2019. Crude dementia incidence rate estimated in seven 4-year subcohorts. Rates are per 1000 person-years. Dementia was defined using algorithmic diagnosis only, excluding doctor diagnoses.

*Age- and sex-standardized rate using England and Wales 2011 Census population estimates

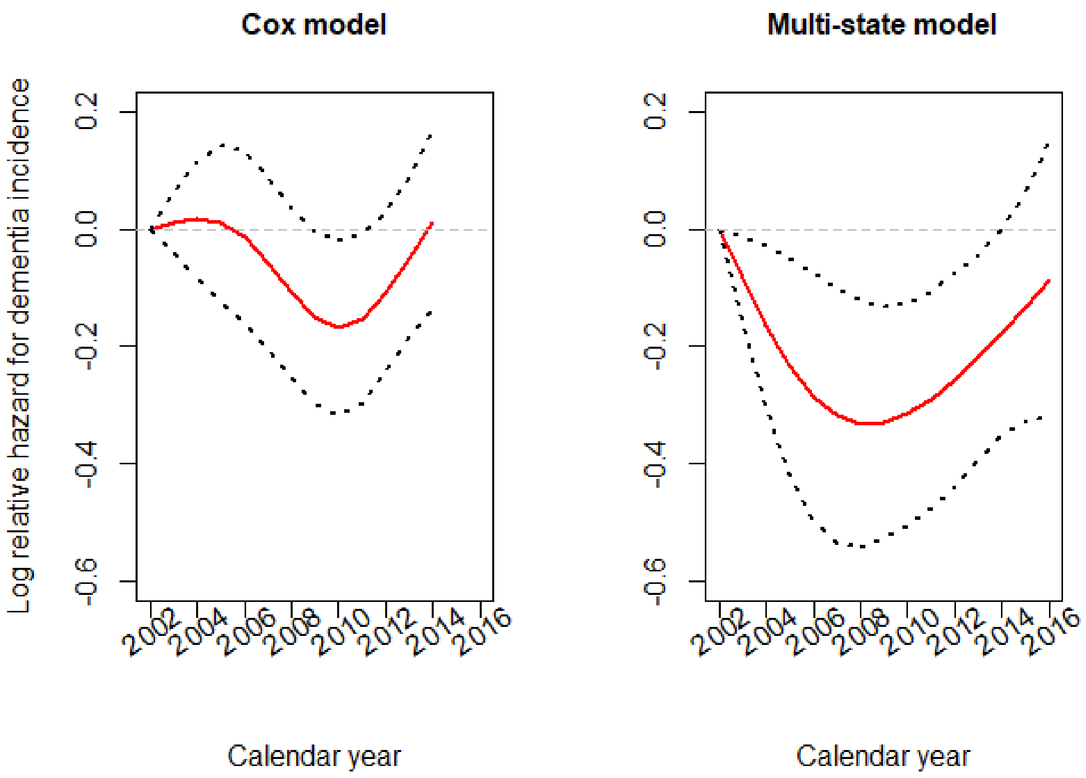

Figure S2 Age and sex adjusted dementia incidence rate ratio (logarithmic scale) estimated from Cox model (A) and multi-state model (B), 2002 to 2019. Reference year 2002. Dashed lines represent 95% confidence intervals. English Longitudinal Study of Ageing.

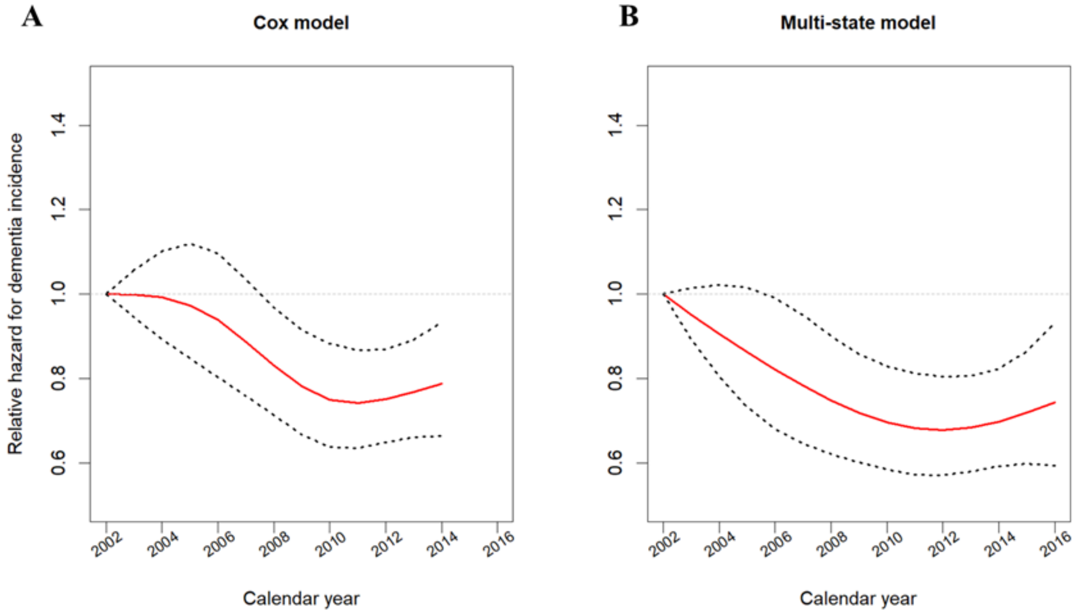

Figure S3 Age and sex adjusted dementia incidence rate ratio estimated from Cox model (A) and multi-state model (B), 2002 to 2019. Reference year 2002. English Longitudinal Study of Ageing. Dementia was defined using algorithmic diagnosis only, excluding doctor diagnoses.

Footnote

Multi-state model (see Figure S1) accounting for potential bias due to deaths between waves among newly incident demented cases. Incidence rate for final wave 2018/2019 cannot be estimated.

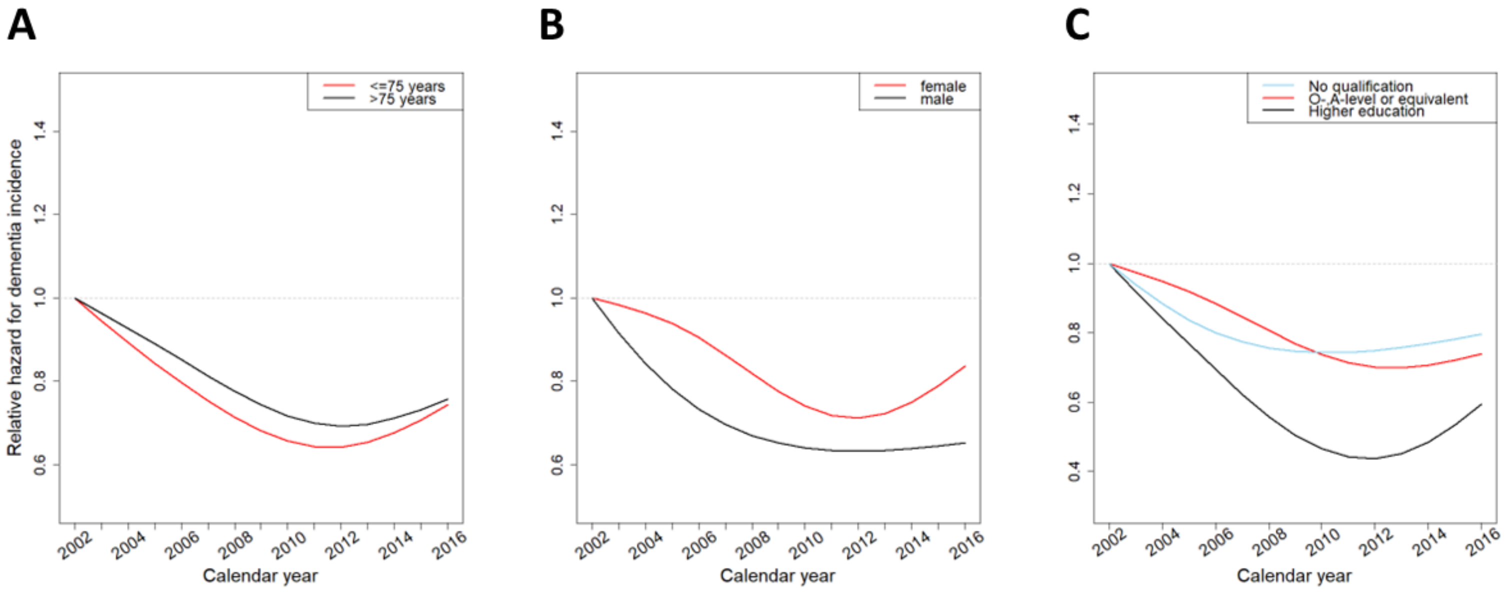

Figure S4 Age and sex adjusted dementia incidence ratio by age (A), sex (B) and education attainment (C)

Footnote

Estimates from multi-state model using English Longitudinal Study of Ageing, 2002-2019. Reference year 2002. P-value for interaction: 0·85 for age, 1 for sex and 0·37 for education attainment. Dementia was defined using algorithmic diagnosis only, excluding doctor diagnoses.

IMPACT-BAM model diagram

Figure S5 presents IMPACT-BAM model structure. Detailed description of the baseline model, states definition, and calculation of transition probabilities have been previously described.[17,32]

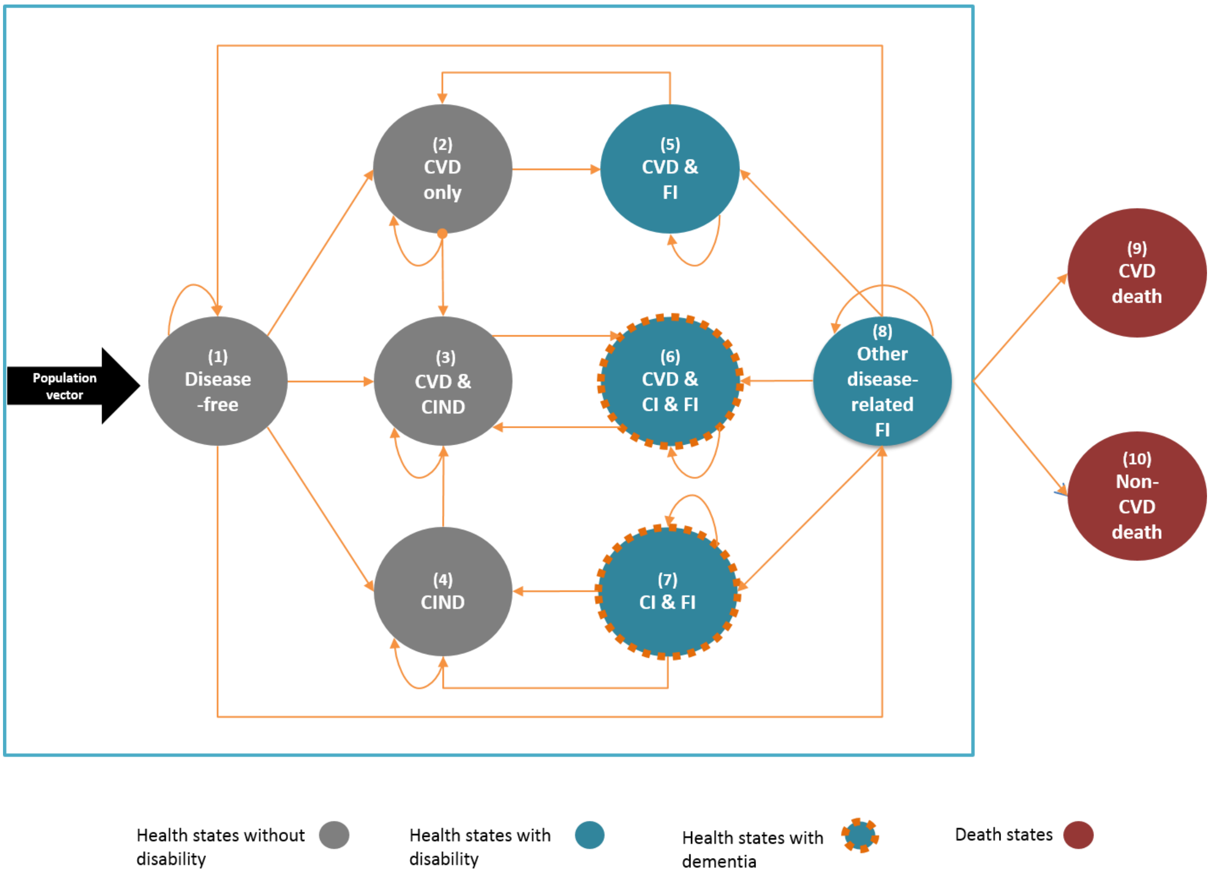

Figure S5 IMPACT-BAM model structure. Transitions to death states 9 and 10 are possible from any state. (Source: Guzman-Castillo et al. Lancet Public Health 2017 supplementary material[32])

Overview of IMPACT Better Aging Model

The IMPACT Better Ageing Model (IMPACT-BAM) is a discrete-time Markov model which follows the progression of a healthy population (aged 35+ years old) from England and Wales into ten different health states characterised by the presence or absence of cardiovascular disease (CVD), cognitive impairment and functional impairment from 2011 to 2040. The model structure is presented in Figure S5, and the health states are described in Table S5.

Input data were updated using nine waves of ELSA data from 2002 to 2019. Prior to simulation, we populated each state in the model based on ONS population estimates in 2011 (start year) and prevalence of the above conditions from ELSA, except for the new cohort of 35-year-olds that enters the system through the disease-free state. The simulation allows individuals to move to other states in the model. The arrows in Figure S5 indicate the possible movements of people between these ten states, which are governed by one-year probabilities of transition. For example, a healthy 55-year man starts the simulation in state 1 (Disease-free state) in 2011. He moves to state 2 (CVD) in 2012 after having a stroke. In 2013 he could either die from complications of the stroke (he moves to state 9), any other causes (he moves to state 10) or he could develop cognitive impairment (moving to state 3) or disability (moving to state 5). As above, movements to any state are driven by transition probabilities. Then, to calculate the number of people with dementia in year t, we count the number of people in the states that represent dementia (states 6 and 7) at year t.

We assume that annual changes in CVD incidence paralleled the annual changes in CVD mortality as observed in ELSA.[17]

Table S5 Description of the health states (Source: Guzman-Castillo et al. Lancet Public Health 2017 supplementary material[32])

**Health state**	**Name**	**Description**
1	Disease-free population	People free of cardiovascular disease (CVD), cognitive impairment (CI) or functional impairment (FI)
2	CVD only	Cardiovascular disease
3	CVD and CIND	Cardiovascular disease and cognitive impairment no dementia
4	CIND	Cognitive impairment no dementia
5	CVD and FI	Cardiovascular disease and functional impairment
6	CVD, CIND, and FI	Cardiovascular disease and dementia (cognitive + functional impairment)
7	Dementia	cognitive + functional impairment
8	Other disease-related FI	Functional impairment no related to CVD or/and Dementia
9	CVD death	Death from CVD causes
10	Non-CVD death	Death from a different cause than CVD

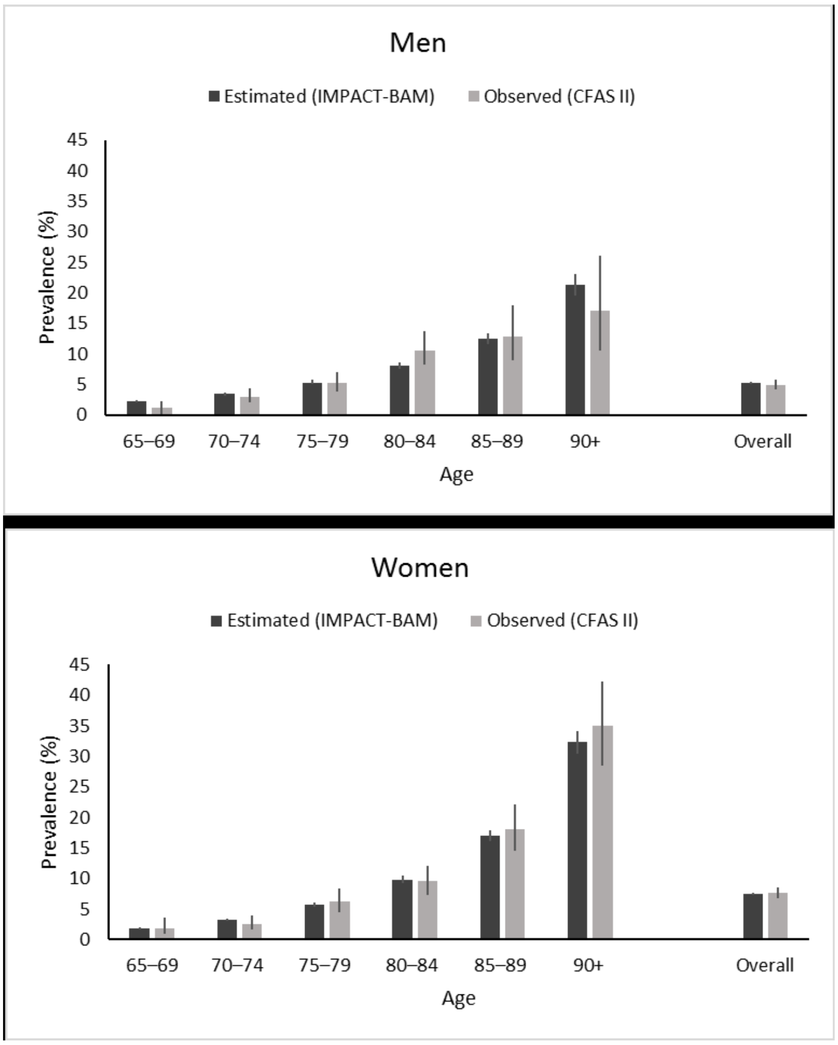

Figure S6 IMPACT-BAM predicted estimates for prevalence of dementia compared with estimates from the Cognitive Function and Ageing Study (CFAS II) in 2011. Error bars represent 95 % uncertainty intervals for estimates from IMPACT-BAM, and 95% confidence intervals for estimates from CFAS II. (Source: Ahmadi-Abhari et al. BMJ 2017 supplementary material[17])

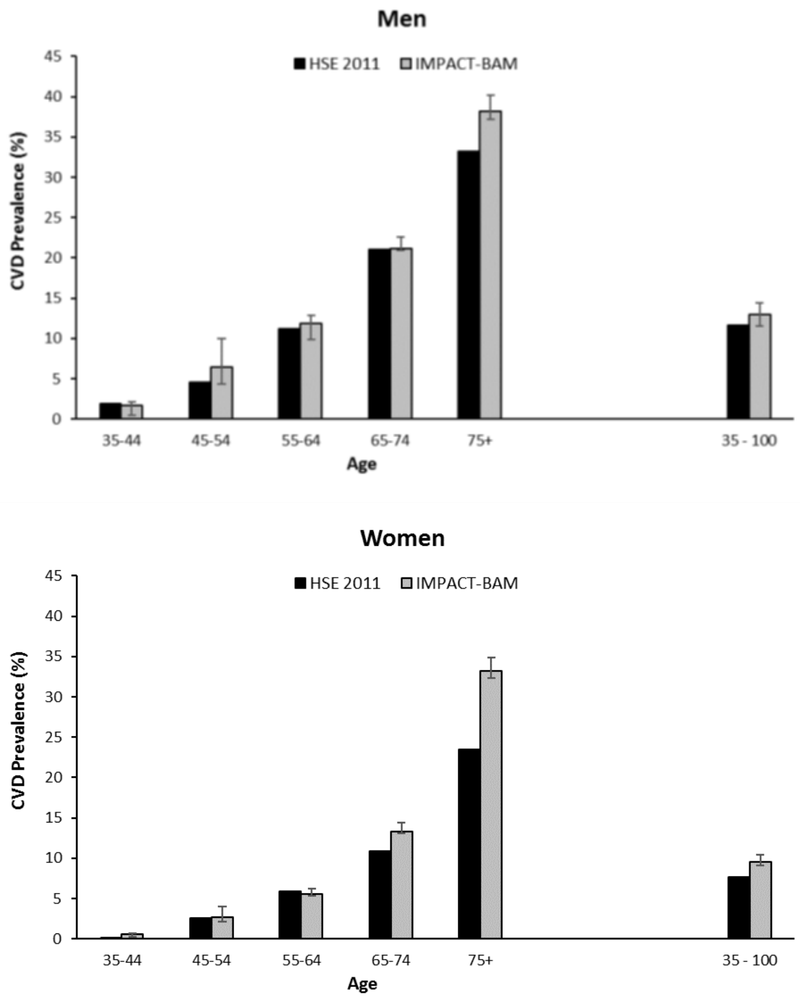

Figure S7 IMPACT-BAM predicted cardiovascular disease prevalence compared with observed estimates from the Health Survey for England in 2011. Error bars represent 95% uncertainty intervals. (Source: Ahmadi-Abhari et al. BMJ 2017 supplementary material[17])

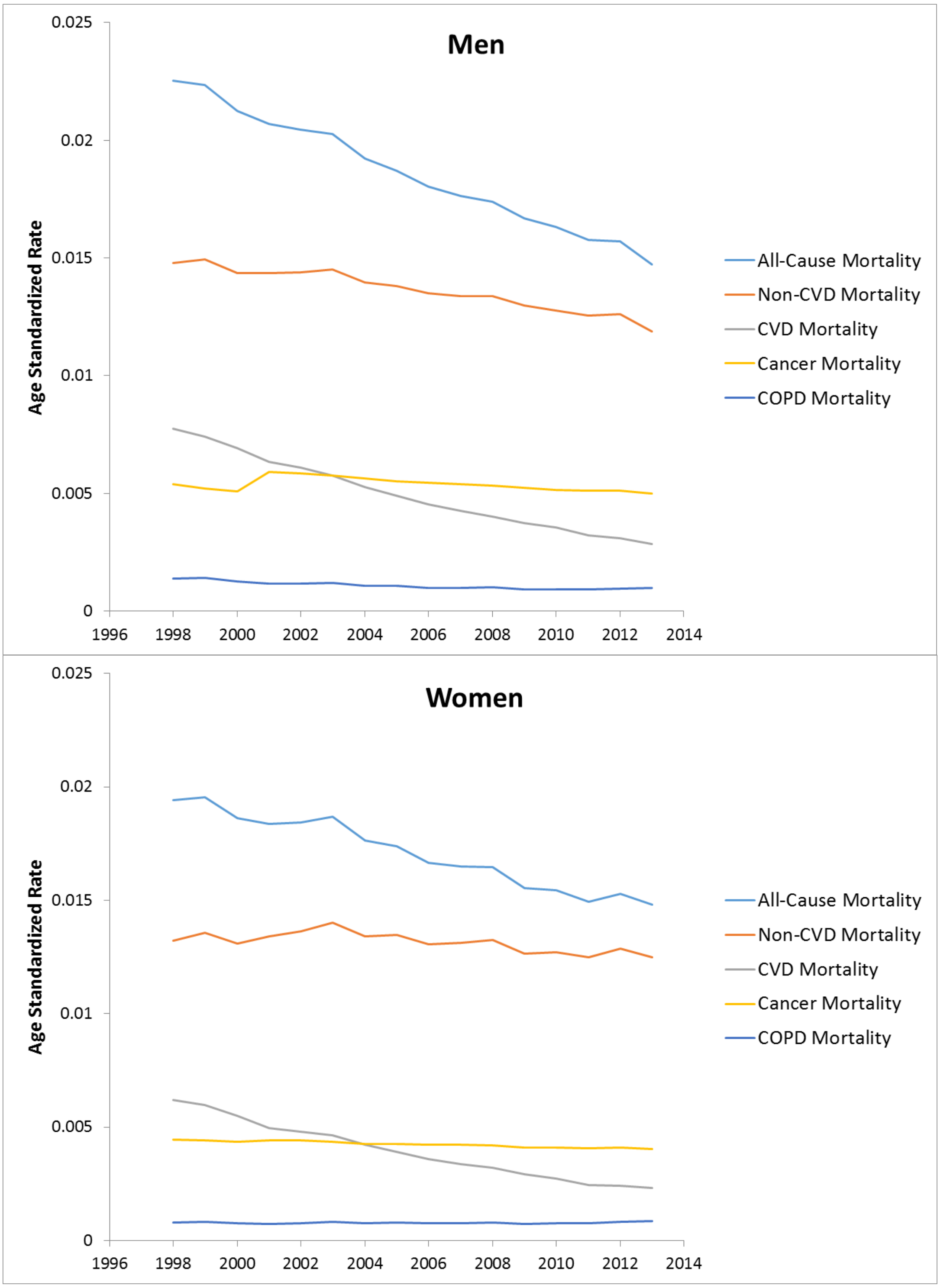

Figure S8 Age-standardised mortality rates by cause, 1998-2013. Data from UK Office for National Statistics. (Source: Ahmadi-Abhari et al. BMJ 2017 supplementary material[17])

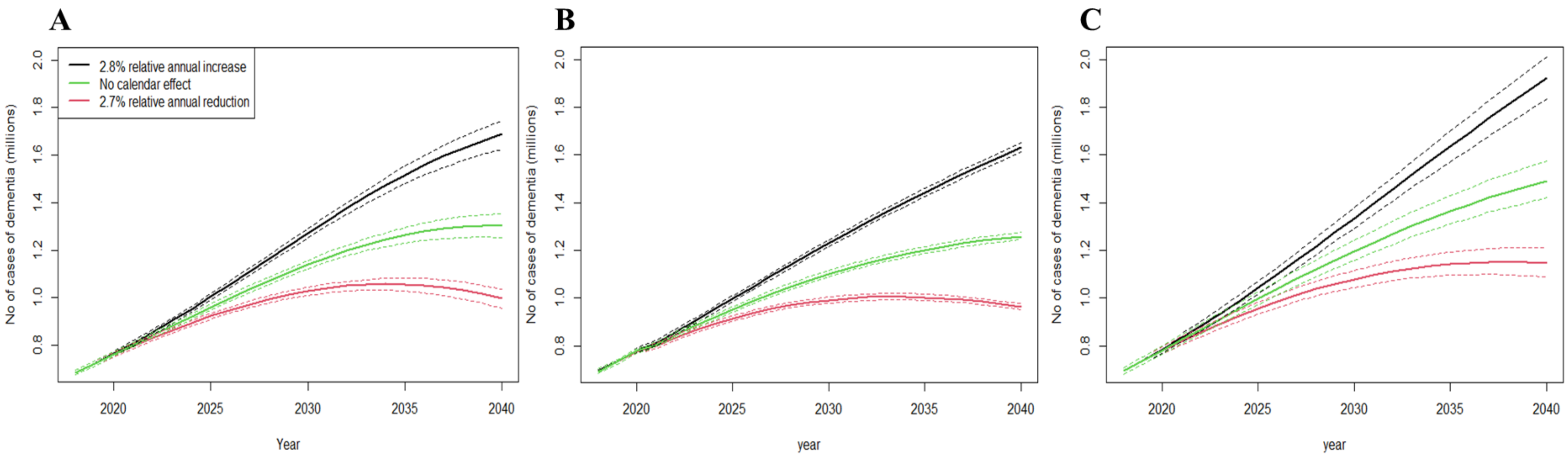

Figure S9 Projected number of people with dementia in England and Wales 2018-2040. Dashed lines represent 95% uncertainty intervals. A: primary scenario, mortality projection was based on two-dimensional P-spline approach; B: conservative scenario, mortality rates from 2018 would persist unchanged to 2040; C: optimistic scenario, mortality projection was based on poisson regression with a log-linear association between calendar year and mortality.

Footnote
2.8% relative annual increase (black line) was estimated using English Longitudinal Study of Ageing (ELSA) 2002-2019 based on a multi-state model accounting for non-linear trend and bias.
2.7% relative annual reduction (red line) was based on previous estimate using ELSA 2002-12

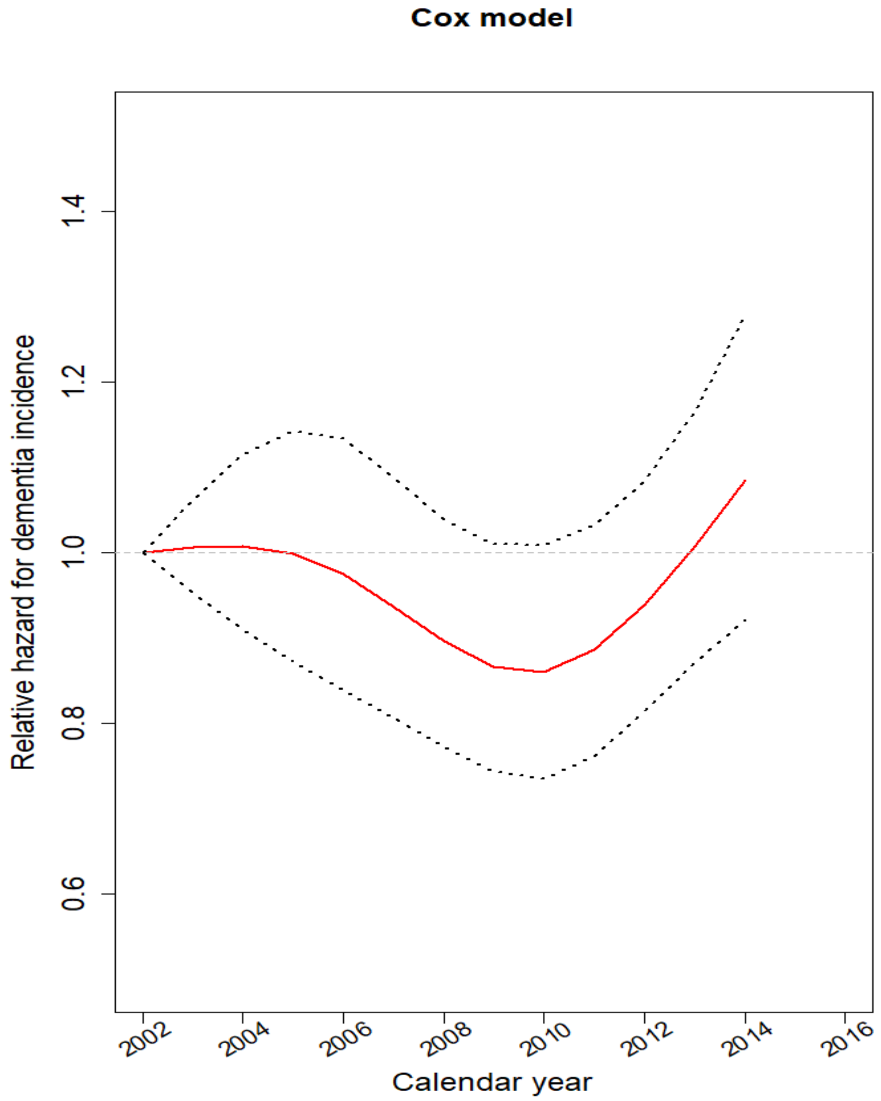

Figure S10 Age and sex adjusted dementia incidence rate ratio estimated from Cox model accounting for sample weight.

References

1 Beerten SG, Helsen A, Lepeleire JD, Waldorff FB, Vaes B. Trends in prevalence and incidence of registered dementia and trends in multimorbidity among patients with dementia in general practice in Flanders, Belgium, 2000–2021: a registry-based, retrospective, longitudinal cohort study. *BMJ Open* 2022; **12**: e063891.

2 Hegelund ER, Mehta AJ, Mortensen LH, Westendorp RGJ. The plasticity of late-onset dementia: A nationwide cohort study in Denmark. *Alzheimers Dement* 2022; **18**: 1287–95.

3 Farina MP, Zhang YS, Kim JK, Hayward MD, Crimmins EM. Trends in Dementia Prevalence, Incidence, and Mortality in the United States (2000-2016). *J Aging Health* 2022; **34**: 100–8.

4 Bohlken J, Riedel-Heller S, Steininger G, Kostev K, Michalowsky B. Trends in Dementia and Mild Cognitive Impairment Prevalence and Incidence in German General and Specialist Practices Between 2015 and 2019. *Journal of Alzheimer's Disease* 2021; **79**: 1683–90.

5 Ding D, Zhao Q, Wu W, *et al.* Prevalence and incidence of dementia in an older Chinese population over two decades: The role of education. *Alzheimer's & Dementia* 2020; **16**: 1650–62.

6 Ding M, Qiu C, Rizzuto D, Grande G, Fratiglioni L. Tracing temporal trends in dementia incidence over 25 years in central Stockholm, Sweden. *Alzheimer's & Dementia* 2020; **16**: 770–8.

7 Power MC, Bennett EE, Turner RW, *et al.* Trends in Relative Incidence and Prevalence of Dementia Across Non-Hispanic Black and White Individuals in the United States, 2000-2016. *JAMA Neurol* 2021; **78**: 1–10.

8 Liu C-C, Li C-Y, Sun Y, Hu SC. Gender and Age Differences and the Trend in the Incidence and Prevalence of Dementia and Alzheimer's Disease in Taiwan: A 7-Year National Population-Based Study. *Biomed Res Int* 2019; **2019**: 5378540.

9 Temporal and Geographic Variation in the Incidence of Alzheimer's Disease Diagnosis in the US between 2007 and 2014 - Kirson - 2020 - Journal of the American Geriatrics Society - Wiley Online Library. https://agsjournals-onlinelibrary-wiley-com.libproxy.ucl.ac.uk/doi/full/10.1111/jgs.16262?af=R (accessed April 16, 2023).

10 Vanderkruk KR, Eberg M, Mahootchi T, Esensoy AV, Seitz DP. Trends of Dementia among Community-Dwelling Adults in Ontario, Canada, 2010–2015. *DEM* 2020; **49**: 286–94.

11 Wolters FJ, Chibnik LB, Waziry R, *et al.* Twenty-seven-year time trends in dementia incidence in Europe and the United States: The Alzheimer Cohorts Consortium. *Neurology* 2020; **95**: e519–31.

12 Taudorf L, Nørgaard A, Islamoska S, Jørgensen K, Laursen TM, Waldemar G. Declining incidence of dementia: A national registry-based study over 20 years. *Alzheimers Dement* 2019; **15**: 1383–91.

13 Petersen MS, Restorff M, Stórá T, Waldemar G, Joensen S. Trend in the Incidence and Prevalence of Dementia in the Faroe Islands. *J Alzheimers Dis* 2019; **71**: 969–78.

14 Rajan KB, Weuve J, Barnes LL, Wilson RS, Evans DA. Prevalence and incidence of clinically diagnosed Alzheimer's disease dementia from 1994 to 2012 in a population study. *Alzheimer's & Dementia* 2019; **15**: 1–7.

15 van den Kommer TN, Deeg DJH, van der Flier WM, Comijs HC. Time Trend in Persistent Cognitive Decline: Results From the Longitudinal Aging Study Amsterdam. *J Gerontol B Psychol Sci Soc Sci* 2018; **73**: S57–64.

16 Donegan K, Fox N, Black N, Livingston G, Banerjee S, Burns A. Trends in diagnosis and treatment for people with dementia in the UK from 2005 to 2015: a longitudinal retrospective cohort study. *The Lancet Public Health* 2017; **2**: e149–56.

17 Ahmadi-Abhari S, Guzman-Castillo M, Bandosz P, *et al.* Temporal trend in dementia incidence since 2002 and projections for prevalence in England and Wales to 2040: modelling study. *BMJ* 2017; **358**: j2856.

18 Ohara T, Hata J, Yoshida D, *et al.* Trends in dementia prevalence, incidence, and survival rate in a Japanese community. *Neurology* 2017; **88**: 1925–32.

19 Gao M, Kuang W, Qiu P, Wang H, Lv X, Yang M. The time trends of cognitive impairment incidence among older Chinese people in the community: based on the CLHLS cohorts from 1998 to 2014. *Age and Ageing* 2017; **46**: 787–93.

20 Cerasuolo JO, Cipriano LE, Sposato LA, *et al.* Population-based stroke and dementia incidence trends: Age and sex variations. *Alzheimers Dement* 2017; **13**: 1081–8.

21 Bussel EF van, Richard E, Arts DL, *et al.* Dementia incidence trend over 1992-2014 in the Netherlands: Analysis of primary care data. *PLOS Medicine* 2017; **14**: e1002235.

22 Noble JM, Schupf N, Manly JJ, Andrews H, Tang M-X, Mayeux R. Secular Trends in the Incidence of Dementia in a Multi-Ethnic Community. *J Alzheimers Dis* 2017; **60**: 1065–75.

23 Satizabal CL, Beiser AS, Chouraki V, Chêne G, Dufouil C, Seshadri S. Incidence of Dementia over Three Decades in the Framingham Heart Study. *N Engl J Med* 2016; **374**: 523–32.

24 Matthews FE, Stephan BCM, Robinson L, *et al.* A two decade dementia incidence comparison from the Cognitive Function and Ageing Studies I and II. *Nat Commun* 2016; **7**: 11398.

25 Grasset L, Brayne C, Joly P, *et al.* Trends in dementia incidence: Evolution over a 10-year period in France. *Alzheimers Dement* 2016; **12**: 272–80.

26 Kosteniuk JG, Morgan DG, O'Connell ME, *et al.* Simultaneous temporal trends in dementia incidence and prevalence, 2005-2013: a population-based retrospective cohort study in Saskatchewan, Canada. *Int Psychogeriatr* 2016; **28**: 1643–58.

27 Doblhammer G, Fink A, Zylla S, Willekens F. Compression or expansion of dementia in Germany? An observational study of short-term trends in incidence and death rates of dementia between 2006/07 and 2009/10 based on German health insurance data. *Alzheimers Res Ther* 2015; **7**: 66.

28 Jørgensen TSH, Torp-Pedersen C, Gislason GH, Andersson C, Holm E. Time trend in Alzheimer diagnoses and the association between distance to an Alzheimer clinic and Alzheimer diagnosis. *Eur J Public Health* 2015; **25**: 522–7.

29 Qiu C, Strauss E von, Bäckman L, Winblad B, Fratiglioni L. Twenty-year changes in dementia occurrence suggest decreasing incidence in central Stockholm, Sweden. *Neurology* 2013; **80**: 1888–94.

30 Schrijvers EMC, Verhaaren BFJ, Koudstaal PJ, Hofman A, Ikram MA, Breteler MMB. Is dementia incidence declining?: Trends in dementia incidence since 1990 in the Rotterdam Study. *Neurology* 2012; **78**: 1456–63.

31 Rocca WA, Petersen RC, Knopman DS, *et al.* Trends in the incidence and prevalence of Alzheimer's disease, dementia, and cognitive impairment in the United States. *Alzheimers Dement* 2011; **7**: 80–93.

32 Guzman-Castillo M, Ahmadi-Abhari S, Bandosz P, *et al.* Forecasted trends in disability and life expectancy in England and Wales up to 2025: a modelling study. *The Lancet Public Health* 2017; **2**: e307–13.
